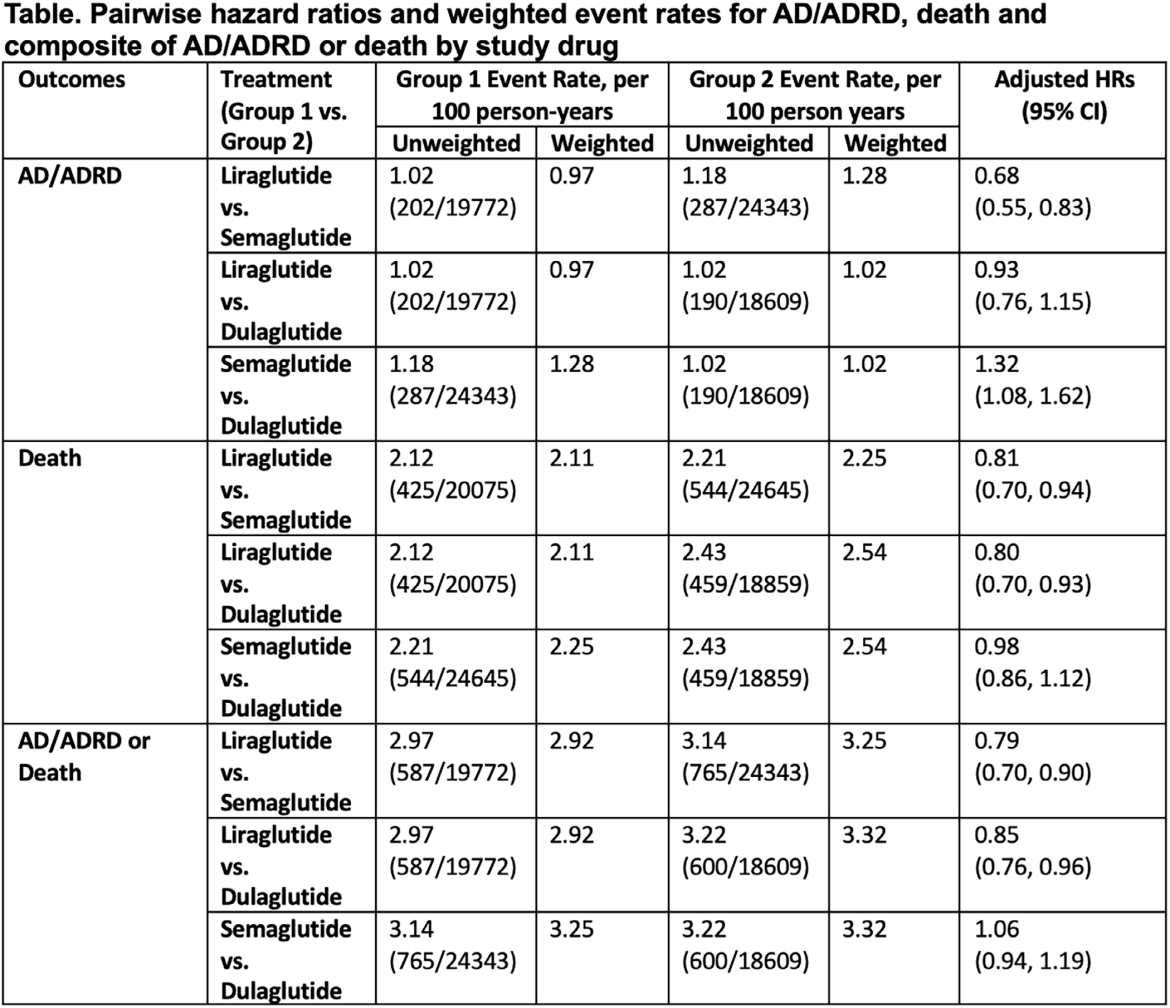# Risk of Alzheimer's Disease and Alzheimer's Disease‐Related Dementias with liraglutide compared to semaglutide and dulaglutide in veterans with type 2 diabetes: An emulated clinical trial observational study

**DOI:** 10.1002/alz70857_101831

**Published:** 2025-12-25

**Authors:** Amara Sarwal, Ravinder Singh, Guo Wei, Sydney E Hartsell, McKenna R Nevers, Niharika Katkam, Augustine Takyi, Akhil R Chakravartula, Jincheng Shen, Srinivasan Beddhu

**Affiliations:** ^1^ University of Utah, Salt Lake City, UT, USA; ^2^ University of Utah, SALT LAKE CITY, UT, USA

## Abstract

**Background:**

Liraglutide, semaglutide and dulaglutide are non‐exendin based GLP‐1RA that are widely used in type 2 diabetes (T2D) management. GLP‐1RA use may be associated with lower risk of dementia, however, intra‐class effects of GLP‐1RA on AD/ADRD risk need to be further elucidated.

**Method:**

We followed the active comparator, new user design to emulate a trial comparing the effect of initiating liraglutide, semaglutide or dulaglutide. Veterans with T2D on metformin who initiated any one of these three agents between 01/01/2018 to 12/31/2021 and did not have baseline dementia, as identified by ICD 9 or 10 codes, were included (*N* = 21,173). Previous use of GLP‐1RA, SGLT2i or insulin glargine was an exclusion criterion. Administrative censor date was 03/31/2023. Generalized propensity score based inverse probability weighting (IPW) was employed to control confounding in the observational data and facilitate comparisons among the three agents. In IPW Cox models with adjustment for baseline covariates, the study drug classes were related to the risk of AD/ADRD, death and composite of AD/ADRD/death.

**Result:**

Of the 21,173 veterans, 25% were initiated on liraglutide, 50% on semaglutide, and 25% on dulaglutide. The mean age was 63 ± 11 years, with 9% female and 17% African American. There were 679 AD/ADRD events over 62,724 person‐years of follow up and 1,428 deaths over 63,579 person‐years of follow‐up. Semaglutide had the highest unweighted event rate of AD/ADRD compared to liraglutide and dulaglutide. In IPW Cox regression models, liraglutide had a lower risk of AD/ADRD compared to semaglutide (HR 0.68, 95% CI 0.55, 0.83), and similar risk compared to dulaglutide (Table). Semaglutide had a higher risk of AD/ADRD than dulaglutide (HR 1.32, 95% CI 1.08, 1.62). In addition, liraglutide had the lowest risk of death as well as composite AD/ADRD/death compared to both semaglutide and dulaglutide.

**Conclusion:**

Liraglutide had a lower risk of AD/ADRD compared to semaglutide as well as a lower risk of death compared to other commonly used GLP‐1RA. Dulaglutide had similar risk of AD/ADRD to liraglutide, however death benefit was not seen.